# Structure and functions of Mer, an innate immune checkpoint

**DOI:** 10.3389/fimmu.2023.1244170

**Published:** 2023-10-23

**Authors:** Eric Ubil, Kashif Rafiq Zahid

**Affiliations:** Department of Microbiology, University of Alabama at Birmingham, Birmingham, AL, United States

**Keywords:** MerTK, macrophage, cancer, immune, MerTK inhibitors, clinical trials

## Abstract

Immunotherapy is a promising therapeutic tool that promotes the elimination of cancerous cells by a patient’s own immune system. However, in the clinical setting, the number of cancer patients benefitting from immunotherapy is limited. Identification and targeting of other immune subsets, such as tumor-associated macrophages, and alternative immune checkpoints, like Mer, may further limit tumor progression and therapy resistance. In this review, we highlight the key roles of macrophage Mer signaling in immune suppression. We also summarize the role of pro-inflammatory (M1) and anti-inflammatory (M2) phenotypes in tumor onset and progression and how Mer structure and activation can be targeted therapeutically to alter activation state. Preclinical and clinical studies focusing on Mer kinase inhibition have demonstrated the potential of targeting this innate immune checkpoint, leading to improved anti-tumor responses and patient outcomes.

## Introduction

Over the last 50 years, the 5-year survival rates of patients with most forms of cancer have improved by more than 15%. However, cancer remains the second leading cause of death in the United States and a leading cause of death worldwide (1). While improvements in diagnosis and treatment have driven increased patient survival, it is clear that further advances are necessary. In recent years, immunotherapies, such as those targeting the adaptive immune response, have improved survival for patients with some forms of cancer (2). Unfortunately, checkpoint blockade has limited efficacy for the treatment of several solid tumors (3, 4) with the success of therapy correlated with overall tumor mutational burden (5). Despite current limits of efficacy, approximately 44% of cancer patients in the United States are eligible for, or have received, immune checkpoint blockade (ICB) therapy. However only 13% of all cancer patients will respond (6). The reasons for the low response rates are still under investigation, but clinical data demonstrates the therapeutic potential of targeting immune checkpoints.

### The role of macrophages in cancer progression

Though innate immune cell targeted therapies lag behind adaptive immunotherapies in the clinic (7), there are well established roles for cells like macrophages in tumor progression. Often one of the most populous intra-tumoral immune cell subsets (8), macrophages are known to adopt pro-inflammatory (M1) or pro-wound healing (M2) phenotypes in the tumor environment. Single cell RNA sequencing has shown that the classical either/or polarization paradigm is not necessarily reflective of the complexity of intra-tumoral polarization states of macrophages, particularly in regard to plasticity and local context. However, an increased presence of M2 macrophages in the tumor environment has been associated with more rapid tumor progression, worse patient outcomes, and increased resistance to adaptive immunotherapy (9). This is problematic because in human malignancies, tumor associated macrophages (TAMs) typically adopt M2 phenotypes and promote cancer progression and metastasis (10). Mechanistically, this can be because M2 TAMs facilitate immune suppression [e.g., through secretions like IL-4, IL-13, CSF-1 and TGF-β (11, 12)] or promote angiogenesis (13). M2 macrophages are also known to remodel the extracellular matrix (ECM) to allow increased tumor growth and metastasis (14). In addition, they have been implicated in reducing the efficacy of adaptive immunotherapy by altering properties of the ECM that allow immune cell infiltration (15, 16). In contrast, M1 macrophages are known to directly limit tumor growth through production of reactive oxygen species or by coordinating the anti-tumor immune response via secretion of inflammatory cytokines (e.g., TNF-α and IFN-γ) (17).

### Mer is an innate immune checkpoint

Macrophages are being actively targeted therapeutically because of their role in orchestrating anti-tumor immune responses (18, 19). One area of intense research is identification of other immune checkpoints. We and others have described Mer (MerTK) (20, 21) as an innate immune checkpoint in macrophages that can be targeted by cancer cells to limit the anti-tumor immune response. However, there are several Mer structure function relationships that have not been fully elucidated. This review describes structural features of Mer, data supporting its roles as an innate immune checkpoint, and pharmacologic therapeutics targeting Mer in the treatment of cancer.

## Structural features of Mer

**Figure 1:**
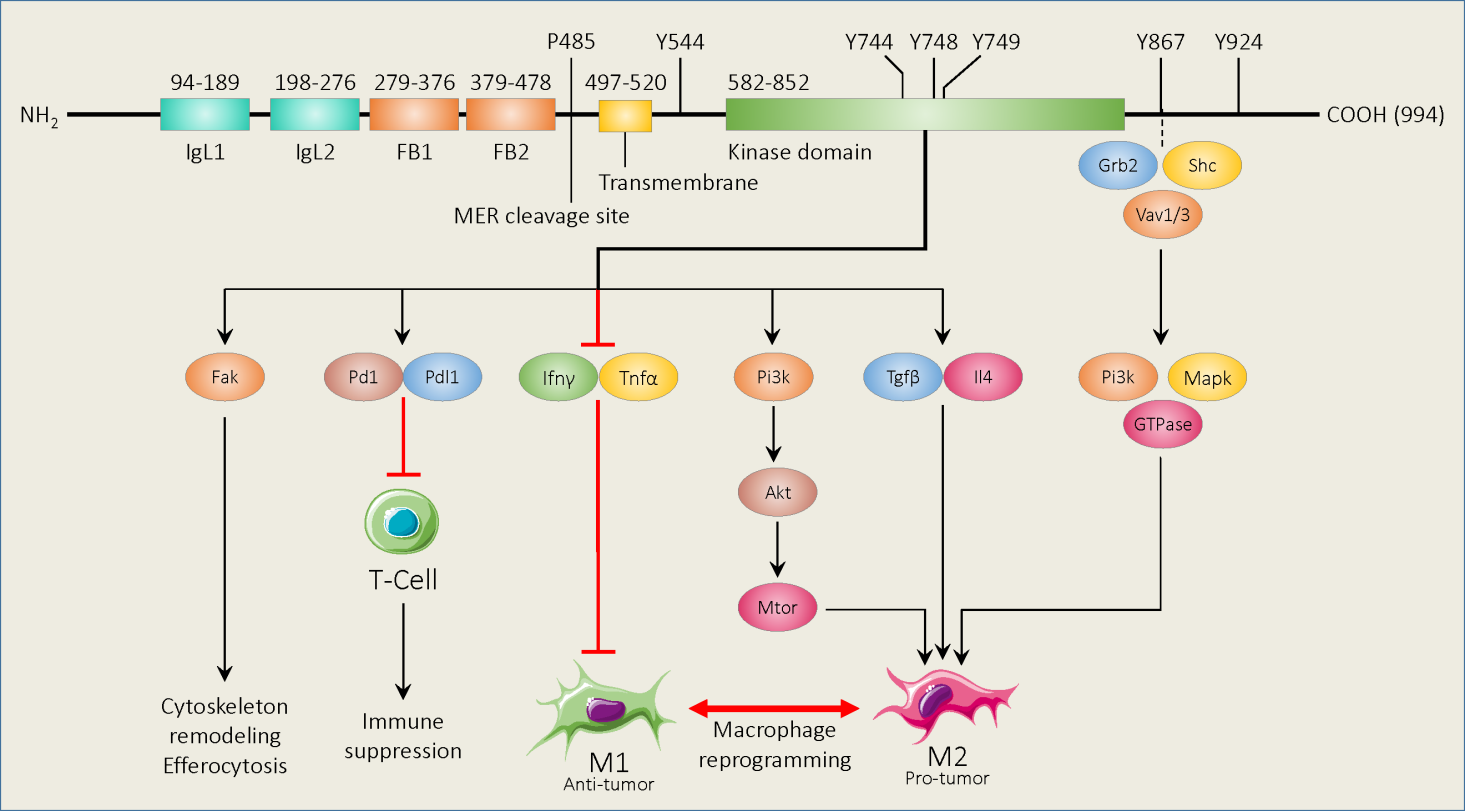
Schematic of murine Mer functional domains and known binding and phosphorylation sites, and downstream signaling pathways. Beginning at the amino terminus, Mer contains 2 immunoglobin-like domains (IgL1,2), 2 fibronectin type III domains (FB1,2), and a transmembrane domain followed by a cytoplasmic kinase region. The Mer cleavage site is found between the second fibronectin type III domain and the transmembrane domain. The Mer adapter binding site is between the kinase domain and the carboxy terminus. Amino acid positions for each functional area are shown above the diagram. Mer activation promotes various cellular processes and immune suppression using the downstream pathways shown. Mer signaling mediated by adapter binding protein binding also promotes a macrophage M2 phenotype.

Mer belongs to the Tyro3/Axl/Mer family of receptor tyrosine kinases (RTKs) and is structurally related to the other receptors. Beginning at the amino terminus, Mer is comprised of 2 immunoglobin-like domains, 2 fibronectin type III domains, and a transmembrane domain followed by cytoplasmic kinase domain (22) (**Figure 1**). Between the second fibronectin type III domain and the transmembrane domain is a cleavage site (**Figure 1**). Cleavage at this site yields a soluble domain capable of acting as a decoy receptor through binding to Mer ligands (23). Elements of Mer are also capable of translocating to the nucleus to perform functions that are still unclear (24). Lastly, Mer has an adapter binding site between the kinase domain and the carboxy terminus (**Figure 1**). While some reports indicate kinase-dependent adapter protein recruitment (25), others demonstrate a potentially kinase-independent role for this protein domain (26). In the following sections, findings related to each Mer domain and their prospective roles in contributing to an innate immune checkpoint are described.

### Activation and effects of the Mer kinase domain

MER binding with known ligands, including Growth arrest specific 6 (Gas6) (27), Protein S (Pros1) (28), Tubby (Tub1) (29), Tubby-like protein 1 (Tulp1) (30), and Galectin 3 (Gal3) (31) can induce autophosphorylation of the tyrosine residues within the activation loop of the kinase domain (Tyr749/53/54, Human) (32). Kinase activation initiates the process of efferocytosis, the engulfment of apoptotic material, through coordinated cytoskeletal rearrangement (33). While ligand binding to apoptotic material promotes efferocytosis, engulfment can still be induced by ligands alone, but efficiency is reduced (34). Studies have also shown that efferocytosis can limit NF-kB signaling in macrophages to reduce inflammatory cytokine expression and limit pro-inflammatory (M1) polarization (35).

Alternatively, efferocytosis has been shown to promote a macrophage pro-wound healing (M2) phenotype, including increased secretion of immune suppressive cytokines (e.g., IL-4/10 and TGF-β).This can downregulate the local and systemic immune responses and allow for tumor immune escape (36). Mer-mediated efferocytosis can also increase immune suppression by causing macrophage upregulation of programmed death-ligand 1 (PD-L1) (37).

Through clearance of apoptotic material, Mer reduces the presence of Damage Associated Molecular Patterns (DAMPs), such as extracellular ATP, endogenous nucleic acids, and transcription factors like HMGB-1 (38), which could potentially activate the M1 response (39, 40). Clearance of tumor cell debris may further deprive macrophages and other antigen presenting cells of tumor antigenic peptides which would otherwise be presented to T cells, either through Major Histocompatibility Complex II (MHCII) to CD4^+^ T cells, or through cross-presentation to CD8^+^ T cells (41, 42). That process may limit potential T cell effector functions and diminish the overall anti-tumor response.

As an example of how both features contribute to immune escape, a recent study shows that Mer-mediated efferocytosis leads to tumor progression and immune tolerance in osteosarcoma by increasing M2 polarization and PD-L1 expression (43).

### Mer can be cleaved to yield a soluble form of the protein

Macrophage membrane-bound Mer can be cleaved at Pro485-Ser486 in murine macrophages (44) by the metalloproteases ADAM10/17 to yield a soluble version of Mer (sMER) (23, 44). Subsequently, cleaved Mer contributes to defective efferocytosis in leukemia (23). After cleavage, sMer can act as a competitive Mer inhibitor and serve as a decoy for its ligand Gas6 (23). Alternatively, sMer can be localized to multiple intra-cellular compartments, including the cytoplasm, the nucleus, and the proteasome (45). While the role of Mer cleavage is not fully understood in the cancer context, the presence of soluble Mer has the potential to add further nuances to TAM signaling.

### Mer can be localized in the nucleus

Mer is generally thought to be maintained at the cell membrane or in endosomes, though some studies have found that Mer can be translocated into nucleus. While not described in macrophages, Borgman et al. showed ligand dependent translocation of MER into the nucleus of human dendritic cells (DCs). Further, the authors found that MER acts as a transcription factor and that the amount of MER in the nucleus is associated with the differentiation state of DCs. Interestingly, the process was also regulated by the transmembrane receptor LRP-1 (46). A similar nuclear translocation phenomenon was observed in cancer cells, though nuclear translocation was determined to be regulated by the phosphorylation of deglycosylated MER, fueling the proliferation and transformation of hepatocellular carcinoma cells (HCC) (47). Similarly, Mer nuclear translocation was observed in leukemic cells (48).

### The adapter binding site can modulate downstream signaling and cytoskeletal rearrangement

More than 20 years ago, Georgescu et al. (49) developed a fusion protein with a CD8 extracellular domain coupled to the Mer intracellular domain in Ba/F3 cells. Point mutations of known Mer phosphorylation sites (i.e., amino acids 544, 614, 825, 867, and 924) were generated within the fusion protein to determine their respective roles in constitutively activated Mer. The study showed that genetic loss of Mer, or mutations of Y867F or Y924F, reduced survival in IL-3 dependent cells cultured in the absence of IL-3. Further analysis identified position 867 as a binding site for the adapter protein Grb2, which is known to mediate Fak, Rac, and PI3K signaling (50). Georegescu and colleagues also determined that Mer 867 was essential for NF-kB activation (48). A later study by Tibrewal et al. (51) conflicted with Georgescu et al. in that NF-kB was not found to be regulated by Mer 867 in Mer mutant transfected RAW264 macrophages. Instead, Tibrewal et al. showed that Gas6 activated Mer 867 mediates PLCγ and Fak signaling and modulated p130 activation (50). Further studies have shown that other SH2 adapter proteins can interact with the intracellular domain of Mer to facilitate cytoskeletal remodeling. One such study showed that Gas6 can induce the release of constitutively bound Vav1 from Mer, to facilitate cellular reorganization for phagocytosis (52). Shelby and colleagues later utilized a screening strategy to identify Mer downstream adapter proteins. Primarily through the study of retinal pigment epithelium, they confirmed that Mer interacts with Grb2, but also with Vav1/3, PIK3R1 and Src to facilitate cellular remodeling (53). Taken together, these studies indicate that, in addition to the Mer kinase domain, there are additional molecular features important to downstream signaling.

## Pharmacologically targeting Mer to improve outcomes

Because of its various roles in tumor immune progression, Mer is actively being targeted to increase cancer patient survival. As described in the following sections, Mer-directed therapeutics are in various stages of development, with some already in clinical trials.

### Mer kinase inhibition promotes better outcomes in preclinical models

Several groups have shown that by pharmacologically targeting Mer kinase activity, efferocytosis and other immune suppressive processes may be reduced and lead to better outcomes in preclinical models. Mer-targeted compounds, such as UNC2250 (54), UNC9253 (55), SA4488 (56), and UNC2025 (57), have been investigated to inhibit Mer kinase activity in different murine tumor models like melanoma, prostate, pancreatic, and breast cancer (26). Zhou et al. have also reported that blockade of efferocytosis in TME mediates a switch from apoptosis/efferocytosis to immunogenic cell death of tumor cells (58).

Hsu et al. investigated the effect of the Mer kinase inhibitor XL092 in various human xenograft murine models. They found that XL092 monotherapy alone or in combination with immune checkpoint inhibitors (ICIs) significantly inhibited tumor growth. XL092 treatment led to decreased tumor cell proliferation and angiogenesis and fostered a less immune-permissive TME, including macrophage reprogramming from M2 to M1 polarization and suppression of efferocytosis (59).

BMS794833, a potent Mer inhibitor, was also found to be very effective in suppressing Mer activation and Mer-induced efferocytosis in an animal model (60). Further, in a preclinical model of glioblastoma, the Mer inhibitor UNC2025 results in induction of an inflammatory macrophage phenotype and reduced the fraction of TAMs expressing the anti-inflammatory marker CD206 in the tumor microenvironment (TME) (61). In addition to TAMs, Mer also regulates other immune cells such as T cells, natural killer cells (NKs), and myeloid derived suppressor cells (MDSCs) (62, 63). In most of these immune cells, Mer plays an immunosuppressive function.

Mer expression has been linked to leukemogenesis and therapy resistance in leukemia models (54, 64). These findings led to the use of Mer inhibitors in the treatment of preclinical leukemia models (65). For example, Mer kinase inhibition using MRX2843 prevented efferocytosis in a model of acute myeloid leukemia (AML). Results showed that Mer blockade diminished macrophage immunosuppressive traits by reducing expression of M2 markers, including TIM-3, PD-L1, Arginase-1, and CD163, and promoting macrophage polarization toward an antitumor (M1) phenotype by limiting STAT6 phosphorylation. In addition, Mer inhibition increased the production of cytokines involved in T cell activation, such as IFN-β, IL-1β, and IL-18 via NF-kB activation and prolonged leukemia-free survival (66). Taken together, these findings support the potential of Mer kinase inhibition as a means of increasing the anti-tumor immune response.

### Antibody-based approaches to targeting Mer

Multiples groups have targeted the Mer kinase domain using small molecules, whereas other researchers have developed specific Mer-targeted monoclonal antibodies (mAbs) which have been tested in preclinical models (67, 68). Davara et al. investigated the effect of mAbs in a murine breast cancer model to block Mer function and found that mice treated with Mer neutralizing mAb displayed decreased tumor growth and metastasis. Mechanistically, Mer blockade with mAb or Mer knockout led to suppressed efferocytosis, reduced M2 macrophages, and enhanced infiltration of CD8^+^ T cells into tumors, indicating an anti-Mer mAb can foster host antitumor immunity (69). In breast and colon tumor murine models, antibody mediated blockade of Mer phagocytic engulfment of apoptotic cells also results in dramatic induction of Type 1 IFN response and increased antitumor T cells immunity, as well as enhanced efficacy of ICI therapy (58). Anti-Mer-antibody has significantly inhibited tumor growth either as single agent or along with anti-PD-1 (58). In a murine lung cancer model, combination radiotherapy, anti-PD-1 and anti-Mer, led to suppressed tumor growth, enhanced survival rates, and an overall decrease in metastasis by enhancing the population of CD8^+^ CD103^+^ T_RM_ cells at distal tumor sites. Triple therapy also reprogrammed macrophage to the M1 phenotype and stimulated activation of CD8^+^ T cells and NK cells at metastatic sites (70). Preclinical studies reinforce the idea that Mer-blockade, either by targeting the kinase domain or by using mAbs, can improve outcomes, particularly when combined with other therapies like radiation or checkpoint blockade to shift the immunosuppressive TME into a more immunogenic state, thereby inducing the adaptive immune response for long lasting anti-tumor immunity.

### MER is a biomarker of therapy resistance

Likely due to its various roles in suppressing the anti-tumor response, MER has been associated with resistance to anti-cancer therapies. For instance, MER overexpression has been detected in patients receiving chemotherapy or targeted therapy as first-line treatment. In colorectal tumors, MER is a predictive biomarker against MEK1/2 inhibitors resistance (71). For melanoma patients, MER upregulation is associated with resistance to MEK and BRAF inhibitors (72). Osimertinib has been proposed as a front-line targeted therapy for NSCLC patients with either T790M mutations or activating mutations because of superior efficacy and enhanced overall survival compared with earlier generation *EGFR* tyrosine kinase inhibitors (TKIs), such as erlotinib or gefitinib (73). Unfortunately, osimertinib is facing clinical challenges similar to first generation inhibitors, as only 3% of NSCLC patients showed complete response with osimertinib treatment while most of the patients exhibited residual tumor (74). A recent study has identified MER as a driver of osimertinib resistance and residual tumor growth in *EGFR^MT^* NSCLC patients. These findings showed that MER and its ligand GAS6 are upregulated in *EGFR^MT^* tumors following osimertinib treatment in both the NSCLC patients samples and xenograft models (75).

### Utilizing MER kinase inhibition to treat cancer patients

Supported by preclinical findings, both MER specific and multi-kinase targeted therapies that also target MER are currently in clinical trials for the treatment of cancer. Clinical trials using MER selective inhibitors such as INCB081776 (NCT03522142) (76), ONO-7475 (NCT03176277) (77), and MRX-2843 (NCT03510104) (78) are being conducted for the treatment of different cancers. The selective inhibitor ONO-7475, which targets MER, AXL, and FLT3, is in Phase I clinical trials for advanced solid tumors and acute leukemia after suppressing progression of both forms of cancer in preclinical models (79). INCB081776 is a potent inhibitor of AXL and MER which is being tested along with nivolumab in advanced solid tumors (NCT03522142) (80).

While targeting of MER using small molecular inhibitors has shown clinical promise, issues with drug toxicity and off-target effects have limited their clinical application. Cabozantinib is a multi-kinase inhibitor that has also shows potent activity against AXL and MET (81). Cabozantinib treatment was found to increase hemoglobin levels in ovarian and prostate cancer patients (82, 83). Some patient deaths were also associated with cabozantinib treatment (84).

He et al. conducted the Phase I clinical trial (NCT01285037) of multi-kinase inhibitor merestinib (LY2801653), which also targets MER, to investigate the therapeutic effect and safety of this novel agent in advanced tumor patients. In this study, the authors tested the combination therapy of merestinib along with other therapeutic agents, including cisplatin, gemcitabine, and cetuximab. Results showed that 32% of tumor patients responded to this therapeutic agent and a 120 mg daily dose of merestinib demonstrated a tolerable safety profile and significant antitumor activity (85). CT053PTSA is a multi-kinase inhibitor being tested in a Phase I clinical trial (NCT04577703) in advanced solid tumor patients. Research findings of the trial suggested that CT053PTSA is well tolerated and safe for patients. This Phase I clinical trial has some limitations in that it was performed at a single center and that all the participants were Chinese, which might affect the generalizability of research findings to a broader population (86). Sitravantinib is a multi-kinase novel inhibitor which potentiates ICI therapy by modulating innate and adaptive immune cell changes in TME, thereby significantly improving efficacy of ICI therapy (87). Sitravantinib works partly by reprogramming M2 macrophages to the M1 phenotype (88). A Phase II clinical trial (NCT02954991) of sitravatinib with the immune checkpoint inhibitor nivolumab was conducted in nonsquamous NSCLC patients who experienced disease progression following ICI therapy or chemotherapy (89). Combination therapy of sitravatinib with nivolumab showed a tolerable safety profile and significant antitumor activity and also improved the survival of ICI therapy experienced NSCLC patients. This study has some limitation because it is based on single arm-design, which precluded tumor patients’ randomization to standard or care.

MER inhibitors possess complex and pleotropic action in human tumors and their therapeutic potential depends on local immune microenvironment, mutation burden, tumor type, and drug resistance. For example, a Phase 1/1b clinical trial of sitravatinib monotherapy demonstrated modest clinical outcomes in advanced NSCLC patients (NCT02219711) (90). Alternatively, the Phase 1 SNOW window-of-opportunity clinical trial of combining sitravatinib with nivolumab for treatment of oral cavity squamous cell carcinoma tumor patients demonstrated a tolerable safety profile and meaningful clinical and pathological outcomes (NCT03575598) (91). Importantly, combining sitravatinib with nivolumab resulted in a decreased immunosuppressive TME and led to macrophage reprogramming toward an M1 phenotype in tumor patients who responded to combination therapy (92).

A summary of ongoing clinical trials is presented in **Table 1**.

**Table 1:** Clinical trials investigating MER inhibitors for the treatment of various malignancies.

Drug Name	MER Region Targeted	Additional Targets	Tumor Type	Phase	Trial ID
MRX-2843	Kinase	FLT3	Non-small cell lung cancer Advanced solid tumors Acute myeloid leukemia, Acute lymphoblastic leukemia, Mixed phenotype acute leukemia	I I I	NCT04762199 NCT03510104 NCT04872478
ONO-7475	Kinase	AXL	Acute myeloid leukemia	I	NCT03176277
BMS777607	Kinase	AXL	Triple-negative breast cancer	I/II	NCT02903914
PF-07265807	Kinase	AXL	Renal cell carcinoma	I	NCT04458259
LY2801653	Kinase	AXL, FLT3	Multiple solid tumors	I	NCT01285037
INCB081776	Kinase	AXL	Advanced solid tumors	I	NCT03522142
CT053PTSA	Kinase	AXL, MET, FLT3	Advanced solid tumors	I	NCT04577703

### Potential challenges and alternative benefits of MER kinase inhibitors

MER inhibition has the possibility of on-target off-tumor effects because of the receptor’s role in tissue repair, platelet aggregation, and innate immune regulation. A previous study reported that Mer inhibition by using small molecule inhibitor UNC-569 causes ultra-structural changes in the mouse retina (93). Moreover, MER mutations are associated with human retinitis pigmentosa in various family cohorts (94), suggesting that kinase inhibition may have an effect on vision as well as cancer.

As described previously, MER kinase inhibitors can also have effects on other targets including FLT3, AXL, and MET (**Table 1**). In some instances, these secondary targets can lead to increased drug efficacy as they affect other clinically relevant features of cancerous cells. For instance, FLT3 is often mutated in hematologic malignancies like acute myeloid leukemia (95) and is a drug target in its own right (96). Similarly, AXL has been shown to confer selective advantage to cancerous cells in tumor progression (97) and simultaneously targeting AXL and MER could show increased benefit for patients. Because of structural similarities between MER, AXL, and TYRO3, it can be challenging to develop selective MER inhibitors (98). While relatively less is known about the role of TYRO3 in cancer, targeting MER and AXL may be advantageous.

## Conclusion

With multiple known ligands, structural similarity to Tyro3 and Axl, and several functional domains that can modify downstream functions, Mer signaling is rich in complexity. However, preclinical and clinical studies focusing primarily on MER kinase inhibition have demonstrated the potential to target MER as an innate immune checkpoint, thereby improving the anti-tumor response and patient outcomes. While it is difficult to fully disaggregate the effects of Mer inhibition in immune cells from those taking place concurrently in tumor cells, reviewed elsewhere (99), Mer remains a promising therapeutic target for anti-cancer therapy alone, or in combination with other immunotherapies.

## Author contributions

KZ and EU jointly wrote and edited the manuscript. KZ prepared the figure and table. All authors contributed to the article and approved the submitted version.
